# Altered static and dynamic functional connectivity in major depressive disorder accompanied by high anxiety: evidence from the REST-meta-MDD consortium

**DOI:** 10.3389/fpsyt.2025.1539702

**Published:** 2025-06-09

**Authors:** Lujun Li, Zhijun Zeng, Yaling Zhou, Jinfei Lin, Jiayuan Li

**Affiliations:** ^1^ Chengdu Southwest Children's Rehabilitation Hospital, Chengdu, China; ^2^ The Second Affiliated Hospital of Chengdu Medical College, China National Nuclear Corporation 416 Hospital, Chengdu, China

**Keywords:** major depressive disorder, anxiety, default model network, functional connectivity, fMRI

## Abstract

**Background:**

Major depressive disorder (MDD) is a prevalent mental health condition characterized by persistent low mood, diminished interest in pleasurable activities, and anhedonia. Some patients with depression experience high levels of anxiety, complicating clinical treatment. However, the underlying pathological mechanisms remain unclear.

**Methods:**

The sample comprised 178 participants, including 73 MDD with high anxiety symptom subjects, 55 MDD with low anxiety symptom, and 50 healthy controls registered from multiple sites based on the REST-meta-MDD Project in China. Resting-state functional magnetic resonance imaging (rs-fMRI) data were recorded. Large-scale static and dynamic functional connectivity analyses were conducted to identify specific brain connectivity distinguishing MDD with low and high anxiety symptoms.

**Results:**

While MDD patients with high and low anxiety symptoms exhibit overlapping alterations in dynamic functional connectivity between the auditory cortex and nodes of the salience network, their distinct clinical profiles may be associated with differential functional connectivity patterns between the components of the default mode network (DMN) and the visual network (VN), as well as between the components of the basal ganglia network (BGN) and VN.

**Conclusion:**

The VN–DMN–BGN functional circuit may help elucidate the underlying pathological mechanisms associated with varying levels of anxiety in depressive disorders. Understanding this neural correlation could contribute to the development of targeted therapeutic strategies for MDD.

## 1 Introduction

Major depressive disorder (MDD) is one of the most common mental disorders and is a highly heterogeneous condition characterized by depressed mood, loss of interest or pleasure, and anhedonia (1). Understanding the neuropathological mechanisms of MDD could enhance our ability to diagnose and treat the disorder more effectively. Notably, some patients with MDD exhibit significant anxiety symptoms, while others do not experience pronounced anxiety (2). This variability highlights the complexity of the disorder and suggests that different underlying mechanisms may be involved, potentially influencing treatment strategies. However, the biological mechanisms remain unclear. Elucidating these mechanisms is essential for identifying novel targets for clinical intervention in MDD.

Researchers have indicated that MDD may be associated with dysfunction in emotional regulation, involving the core brain regions such as the hippocampus, prefrontal lobe, amygdala, and hypothalamus (3, 4). Abnormalities in the connectivity and activity of these regions may contribute to the impaired emotional and social functioning in individuals with MDD. Additionally, alterations in sensory thresholds may lead to either heightened sensitivity or diminished responsiveness to sensory input, further affecting daily functioning and social interactions (5). Recent evidence also suggests that the visual network is implicated in the neuropathological mechanism of both MDD and anxiety disorder (5–7). Understanding how sensory perception networks contribute to depression may offer valuable insights for developing targeted therapeutic interventions aimed at restoring normal sensory processing and improving emotional regulation (8).

Moreover, MDD is related to mediated neural activity in non-motor regions, such as the nodes within the default mode network (DMN) (9–13). Most interestingly, a recent study indicated that the symptoms of MDD subjects are associated with reduced visual network (VN) and DMN regions during stimulation with especially rapid visual stimuli (13). Different treatment approaches have been shown to modulate GABA levels in the VN in individuals with MDD (14). The visual cortex has also been reported to exhibit globally reduced activity, which is associated with impaired visual psychophysical performance (15, 16). These findings suggest that the DMN and VN may be involved in the neuropathological mechanisms of MDD. However, the distinct neural pathways differentiating MDD with high anxiety symptoms from MDD with low anxiety symptoms remain unclear.

The resting-state functional magnetic resonance imaging (rs-fMRI) has emerged as a powerful tool in clinical neuroscience (17–20). Clinical neuroscience has adopted transdiagnostic methodologies to explore the neurobiological abnormalities of mental disorders (21, 22). A highly synchronized network at the resting state is thought to represent distinct primary sensorimotor, emotional, or cognitive processes. Specifically, static and dynamic functional connectivity are crucial methodologies for investigating brain function and elucidating the pathological mechanisms underlying depression. Static functional connectivity examines the consistent patterns of brain activity over time, providing insights into the stable relationships between different brain regions (23). This approach has revealed altered connectivity in networks such as the DMN in individuals with depression, highlighting disruptions in emotional regulation and self-referential thought processes. In contrast, dynamic functional connectivity captures the temporal fluctuations in brain connectivity, reflecting the brain’s adaptability and responsiveness to changing cognitive and emotional demands (24, 25). Dynamic connectivity analyses can reveal periods of heightened connectivity that correlate with depressive episodes, as well as moments of disconnection that may indicate resilience or recovery. Together, these approaches provide a comprehensive framework for understanding the complex interplay of brain networks in depression (26). By integrating both static and dynamic perspectives, researchers can better identify biomarkers for depression, inform treatment strategies, and ultimately enhance our understanding of MDD.

Based on previous research, we hypothesized that MDD patients with high anxiety symptoms would exhibit distinct functional connectivity (FC) within primary systems, as well as in associated modulatory networks, compared to those with low anxiety symptoms. To test this hypothesis, we conducted large-scale static and dynamic FC analyses using multi-site datasets of patients with MDD. We propose that these differential functional changes may offer insights into the underlying biological mechanisms of MDD.

## 2 Materials and methods

### 2.1 Participants

This study was based on the REST-meta-MDD Project of resting-state fMRI initiated in China (27–29). The fMRI data were downloaded from the website of a public database (rfmri.org/REST-meta-MDD). In the REST-meta-MDD Project, the Hamilton Depression Scale (HAMD) was used to evaluate the depression symptom severity of patients. The severity of anxiety was examined using the Hamilton Anxiety Scale (HAMA) in MDD patients. Depression patients with a HAMA score greater than 14 were defined as the high-anxiety depression (HAD) group, while those with a score less than 14 were defined as the low-anxiety depression (LAD) group. Matched healthy controls (HCs) were also recruited. The sample comprised 178 participants, including 73 MDD with high anxiety symptom subjects, 55 MDD with low anxiety symptom subjects, and 50 healthy controls. Subject demographics are displayed in **Table 1**. All data were identified and anonymized. All subjects provided informed consent in accordance with the requirements of the ethics committee of the local institutional review boards.

**Table 1:** Participants’ fundamental information.

	HAD	LAD	HC	*p*
Gender (male/female)	34/39	27/28	25/25	0.923^a^
Age (years)	34.49 ± 9.96	32.25 ± 11.81	34.06 ± 11.22	0.497^b^
Education (years)	11.31 ± 3.14	10.94 ± 3.82	11.44 ± 3.69	0.749^b^
HAMD	20.47 ± 3.04	20.16 ± 4.92	–	0.655^c^
HAMA	25.52 ± 5.39	10.78 ± 2.93	–	< 0.001^c^

Indicated values are shown as mean ± standard deviation.

HAD, high-anxiety depression disorder; LAD, low-anxiety depression disorder; HC, healthy controls; HAMD, Hamilton Depression Scale; HAMA, Hamilton Anxiety Scale.*^a^* Indicates the p values from the comparison analysis (chi-square test). *^b^* Indicates the p values from the comparison analysis (one * there ANOVA).*^c^* Indicates the p values from the comparison analysis (two sample t test).

### 2.2 Data acquisition and preprocessing

All resting-state fMRI data were preprocessed at each site according to a standardized preprocessing protocol on Data Processing Assistant for Resting-State fMRI (DPARSF) (27). The detailed preprocessing steps were as follows: discarding the first 10 volumes, slice-timing correction, realignment, coregistration, normalization, and nuisance regression. Nuisance signals include the Friston-24 head motion parameters, white matter, and cerebrospinal fluid. Participants with mean framewise displacement (FD) larger than 0.2 were excluded. Finally, a linear trend was included as a regressor to account for drifts in the Blood Oxygen Level-Dependent (BOLD) signal, and temporal band-pass filtering (0.01–0.1 Hz) was applied to all time series.

### 2.3 Atlas-based static and dynamic functional connectivity analyses

**Figure 1:**
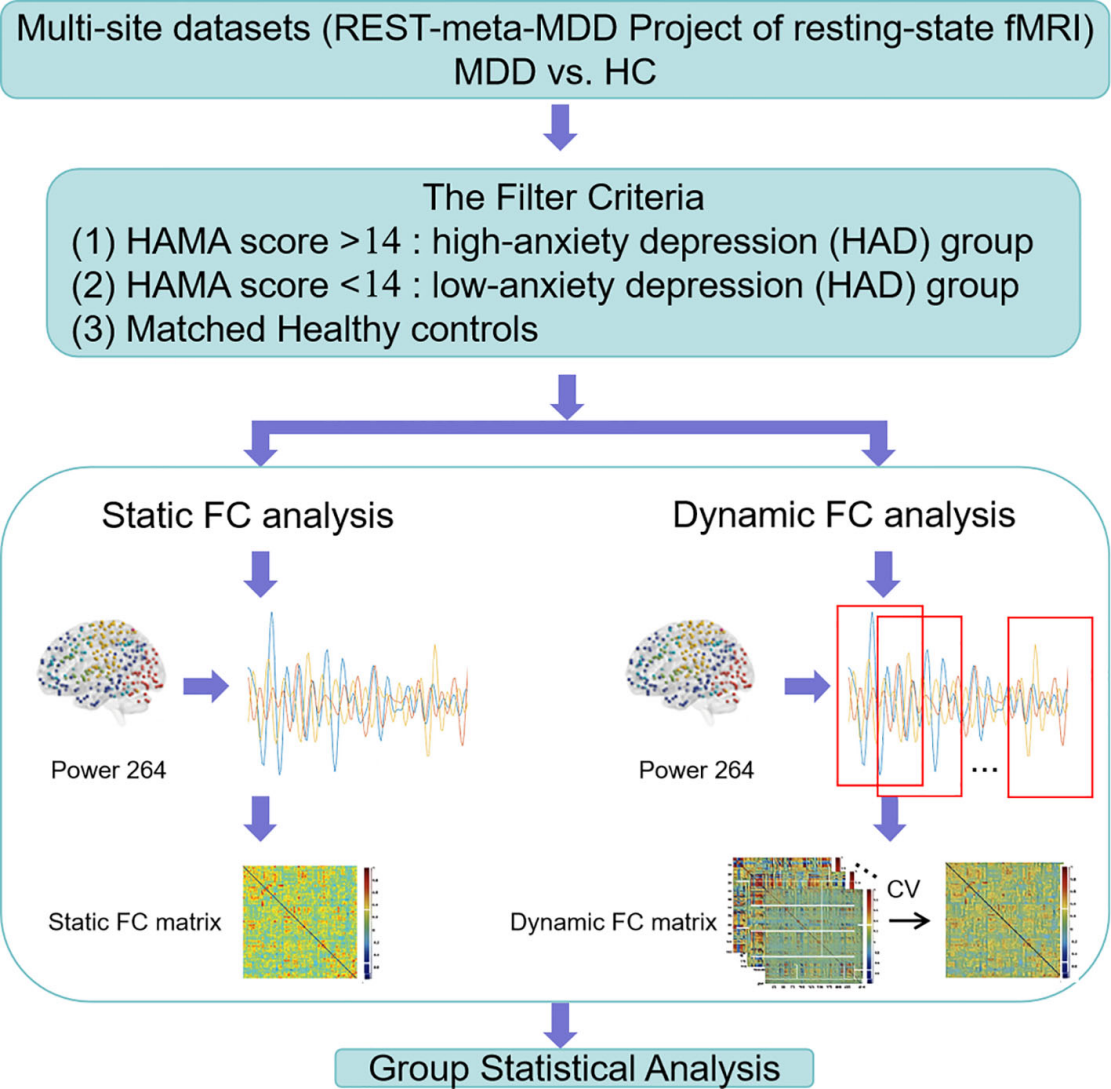
An overview of the analytic steps.

Static and dynamic FC analyses were performed. An overview of the analytic steps is shown in **Figure 1**. For each subject, key nodes within the whole brain were defined based on Power’s 264 functional regions of interest (ROIs). To assess the static and dynamic FC among these regions, a series of steps were performed. Pearson’s correlation coefficients were calculated among 264 ROIs. The resulting values were transformed to approximate a Gaussian distribution using Fisher’s r-to-z transformation. The FC score based on the whole time course was considered as the static (r*_static_*) FC. Dynamic FC was also measured using a sliding window. The time courses were segmented into windows to efficiently capture cognitive status. Previous research has indicated that the minimum window length should be no less than 1/f*_min_.* Thus, the time courses were segmented into 100-s windows, sliding by 2 s of data. Within each window, Pearson’s correlations were computed among 264 ROIs. Across *n* windows, the dynamic FC was defined as the coefficient of variation score in each connectivity.

### 2.4 Statistical analysis

Based on prior studies, ComBat has been recognized as one of the most effective harmonization techniques, as it successfully eliminates site-related unwanted variation while retaining inter-subject biological differences (30, 31). Thus, for the static and dynamic FC, the ComBat method was used to remove site effects. In the ComBat model, the age, sex, education years, and FD were included as covariates. ComBat harmonization analyses were performed using a publicly available Matlab package hosted at https://github.com/Jfortin1/ComBatHarmonization. Then, the ANOVA was performed to assess the difference between static and dynamic FC among the HAD group, the LAD group, and the HC group. The significance threshold of group differences for the ANOVA was set to false discovery rate (FDR)-corrected *p* < 0.05. The different matrix was obtained. Then, the altered FC was extracted for the *post-hoc* analysis through a two-sample t-test between the two groups (uncorrected *p* < 0.05).

### 2.5 Correlations with HAMD and HAMA scores

The relationship was assessed between changed FC and coupling symptoms of depression and anxiety. First, the HAMD value was divided by the HAMA value. This score was defined as the coupling of symptoms of each patient. Then, the partial correlation analysis was calculated between the changed FCs and coupling symptoms in the LAD and HAD groups, with age, sex, education years, and FD as covariates (uncorrected *p* < 0.05).

## 3 Results

### 3.1 Altered static functional connectivity

**Figure 2:**
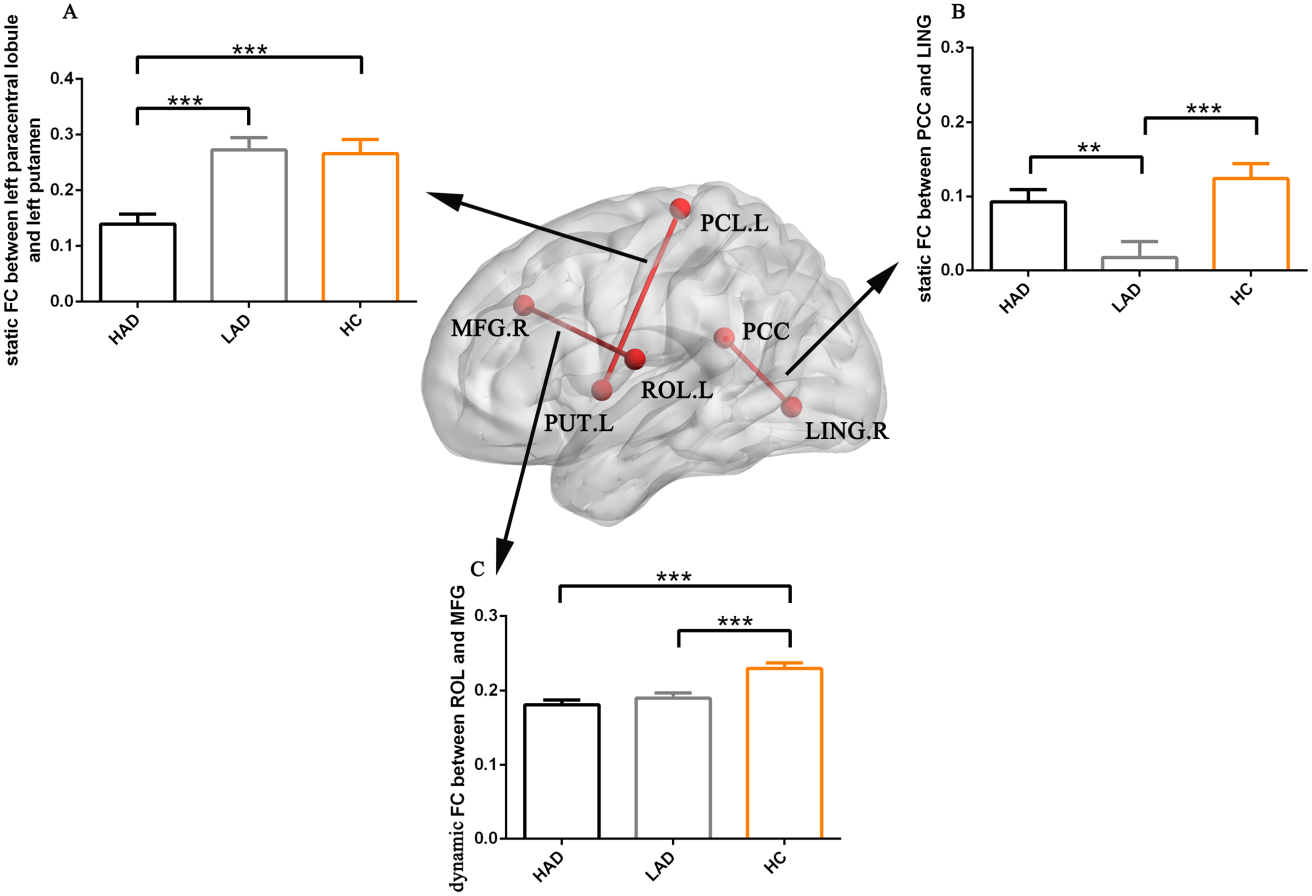
The bar maps represent the *post-hoc* results. The data are expressed as the mean value + standard error. *** *p* < 0.001 and **p < 0.01. **(A)** The difference in static FC between left paracentral lobule and left putamen. **(B)** The difference in static FC between PCC and LING. **(C)** The differences in dynamic FC between ROL and MFG. PCC, posterior cingulate cortex; LING, lingual gyrus; MFG, middle frontal gyrus; ROL, Rolandic operculum; FC, functional connectivity; HAD, high-anxiety depression group; LAD, low-anxiety depression group; HC, healthy control.

The altered static FC was observed between the key node of the dorsal visual network and the subcortical region through ANOVA. *Post-hoc* analysis revealed that the reduced static FC between the left paracentral lobule and putamen within the basal ganglia network (BGN) was observed in the HAD group compared to the HC and LAD groups, but did not show a difference between the LAD and HC groups (**Figure 2A**). Moreover, through ANOVA, altered static FCs were observed between the region of the VN and the region of the DMN. *Post-hoc* analysis found that reduced static FC was observed between the posterior cingulate cortex (PCC) and the lingual gyrus in the LAD group compared to the HAD and HC groups, but did not show a difference between the HC and HAD groups (**Figure 2B**).

### 3.2 Altered dynamic functional connectivity

The abnormal dynamic FC was found between the region of the auditory network (AN) and the region of the salience network (SN). *Post-hoc* analysis found high static FC between the left Rolandic operculum and right middle frontal gyrus in the HC group compared to the HAD and LAD groups, but did not show a difference between the LAD and HAD groups (**Figure 2C**).

### 3.3 Relationship among altered functional connectivities

**Figure 3:**
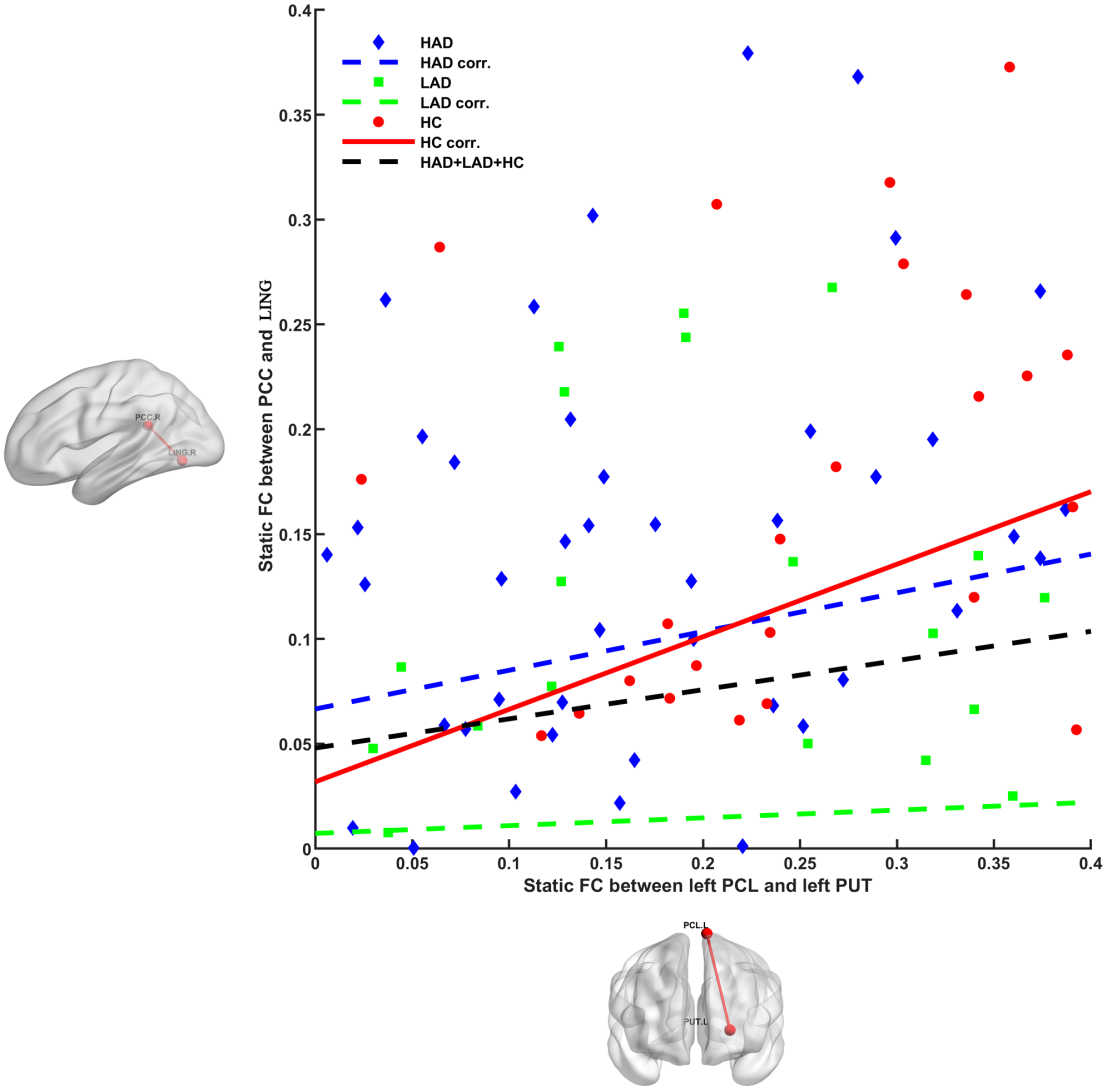
Different relationships between PCL–PUT static FC and PCC–LING static FC in HAD, LAD, and HC groups. Positive relationship was observed in HC group (*r* = 0.434, *p* = 0.0016), whereas this correlation was not apparent in the LAD (*r* = 0.037, *p* = 0.7835) and HAD (*r* = 0.201, *p* = 0.0879) groups. PCL, paracentral lobule; PUT, putamen; PCC, posterior cingulate cortex; LING, lingual gyrus; HAD, high-anxiety depression group; LAD, low-anxiety depression group; HC, healthy control.

Using a *post-hoc* analysis to determine the relationship among altered FCs (**Figure 3**), in the HC group, we observed a positive relationship (*r* = 0.434, *p* = 0.0016) between static FC (left paracentral lobule and left putamen) and static FC (PCC and lingual gyrus), whereas this correlation was not apparent in the LAD (*r* = 0.037, *p* = 0.7835) and HAD (*r* = 0.201, *p* = 0.0879) groups. This is the common deficient functional coupling within the VN–DMN–BGN circuit in the LAD and HAD groups.

### 3.4 Relationship between altered FC and coupling score of HAMD and HAMA

**Figure 4:**
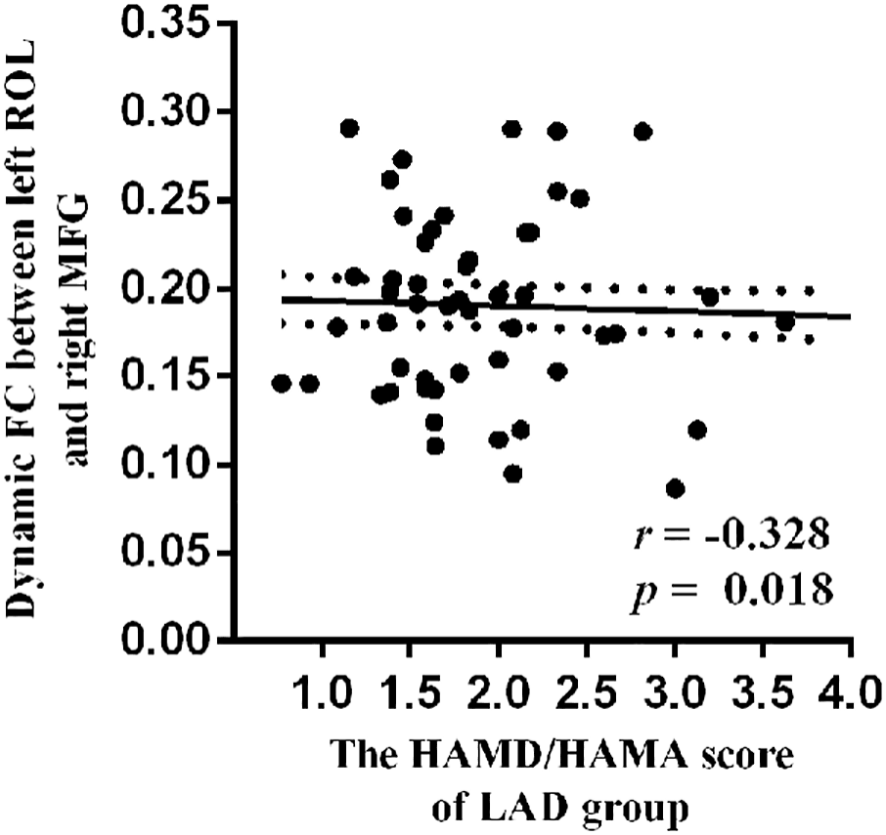
The negative relationship between HAMD/HAMA score and dynamic ROL–MFG FC in LAD group. ROL, Rolandic operculum; MFG, middle frontal gyrus; FC, functional connectivity; LAD, low-anxiety depression group.

A negative relationship (*r* = −0.328, *p* = 0.018) was observed between HAMD/HAMA score and dynamic Rolandic operculum–middle frontal gyrus (ROL–MFG) FC in the LAD group (**Figure 4**).

## 4 Discussion

In this study, we applied static and dynamic large-scale functional connectivity analyses to multi-site MDD datasets. While both MDD patients with high anxiety and those with low anxiety symptoms exhibited overlapping alterations in dynamic FC between the auditory region and nodes of the salience network, their distinct characteristics may be attributed to differences in FC between regions of the DMN and the VN, as well as between the putamen and the paracentral lobule. Elucidating the relationships between specific patterns of aberrant FC and MDD may enhance our understanding of the neuropathophysiological mechanisms underlying the symptom heterogeneity of this complex disorder.

The first main finding is the reduced FC between the putamen and the paracentral lobule, a key region within the dorsal visual pathway, in MDD patients with high anxiety symptoms. The dorsal visual pathway is involved in spatial awareness and motion processing (32). MDD has been associated with reduced FC in visual processing regions, which has been linked to difficulties in visual attention and the interpretation of emotional cues (33). These abnormalities may contribute to the cognitive and emotional symptoms of depression, influencing how individuals perceive and interact with their environment. Conversely, research has shown that individuals with anxiety disorders often exhibit heightened sensitivity to visual stimuli, which may be reflected in altered FC patterns between the visual network and the basal ganglia network (34, 35). In this study, altered static FC was observed in MDD patients with high anxiety symptoms between regions of the dorsal visual network and nodes within the BGN. This difference was not observed in MDD patients with low anxiety symptoms. Together, our findings highlight that abnormal FC between the VN and BGN may be a key feature associated with the neuropathological mechanisms of MDD in individuals with high anxiety symptoms.

Our next key finding consists of the decreased FC between PCC and the lingual gyrus in MDD subjects with low anxiety symptoms. Previous research has indicated that the symptoms of MDD may be related to a psychomotor source with neural changes outside motor regions, for example, regions within the DMN (36) and regions within the visual network (37–39). The DMN is commonly active during rest, and self-referential thought (40) shows increased activity patterns in depressed patients (41, 42). Studies using fMRI have demonstrated that the lingual gyrus, which is associated with processing social and emotional information, exhibits increased connectivity with the occipital lobe (43, 44). This enhanced connectivity may reflect a maladaptive neural response in depression with low anxiety symptoms, where the integration of emotional and visual information becomes dysregulated. In contrast, individuals with anxiety disorders often show reduced DMN–frontoparietal network (FPN) connectivity, which may reflect a reduced state of vigilance and a tendency to focus on external threats rather than internal thoughts (45). Finally, our findings are well in line with the previous finding of decreased local and global synchronization of the visual cortex in MDD (39). In this study, reduced static FC was observed between the PCC and lingual gyrus in the LAD group compared to the HAD and HC groups, but did not show a difference between the HC and HAD groups. Therefore, reduced PCC–lingual gyrus connectivity may represent a specific feature of MDD patients with low anxiety symptoms.

Despite delineating several critical observations, there are several limitations that should be considered. First, the causality between altered functional networks and patient symptoms was not examined in this study. Second, participants did not complete any emotion-related task-based fMRI assessments. Finally, we acknowledge that validation using independent datasets, particularly from our own clinical center, is crucial to confirm the robustness of our results and will be a key focus of our future work.

## 5 Conclusion

Using a data-driven approach with multi-site datasets, the present study provides robust evidence for different DMN–VN and VN–BGN FCs between MDD with low anxiety symptoms and MDD with high anxiety symptoms, supporting a sub-categorization of depression. These networks create an interplay of VN–DMN–BGN functional circuit, which may contribute to our understanding of the neuropathophysiological mechanisms underlying the heterogeneity of symptoms in this complex disorder.

## Data Availability

The original contributions presented in the study are included in the article/Supplementary Material. Further inquiries can be directed to the corresponding author.
